# Roles of **γ** **δ** T Cells in the Pathogenesis of Autoimmune Diseases

**DOI:** 10.1155/2013/985753

**Published:** 2013-02-28

**Authors:** Dinglei Su, Minning Shen, Xia Li, Lingyun Sun

**Affiliations:** ^1^Department of Immunology and Rheumatology, The Affiliated Drum Tower Hospital of Nanjing University Medical School, Nanjing 210008, China; ^2^Department of Rheumatology and Immunology, Nanjing First Hospital, Nanjing Medical University, Nanjing 210006, China

## Abstract

**γ** **δ** T cells are a minor population of T cells that express the TCR **γ** **δ** chains, mainly distributed in the mucosal and epithelial tissue and accounting for less than 5% of the total T cells in the peripheral blood. By bridging innate and adaptive immunity, **γ** **δ** T cells play important roles in the anti-infection, antitumor, and autoimmune responses. Previous research on **γ** **δ** T cells was primarily concentrated on infectious diseases and tumors, whereas their functions in autoimmune diseases attracted much attention. In this paper, we summarized the various functions of **γ** **δ** T cells in two prototypical autoimmune connective tissue diseases, that is, SLE and RA, elaborating on their antigen-presenting capacity, secretion of proinflammatory cytokines, immunomodulatory effects, and auxiliary function for B cells, which contribute to overproduction of proinflammatory cytokines and pathogenic autoantibodies, ultimately leading to the onset of these autoimmune diseases. Elucidation of the roles of **γ** **δ** T cells in autoimmune diseases is not only conducive to in-depth understanding of the pathogenesis of these diseases, but also beneficial in providing theoretical support for the development of **γ** **δ** T-cell-targeted therapy.

## 1. Introduction

*γδ* T cells are a minor population of T cells that express the TCR *γδ* chains. Based on different TCR *γδ* chain expression, human *γδ* T cells can be divided into two subsets: V*δ*1 + T cells that are mainly distributed in epithelial and mucosal surfaces, and V*δ*2 + T cells that generally coexpress V*γ*9 and exist primarily in the peripheral blood and lymphatic system. In normal human peripheral blood, *γδ* T cells, 70–90% of which are V*γ*9V*δ*2 T cells, account for about 1–5% of total T cells, activated by small nonpeptide phosphoantigens (e.g., isopentenyl pyrophosphate (IPP)) in a TCR-dependent and MHC-not-limited manner [1]. In the early stage of immune responses, *γδ* T cells may bridge innate and adaptive immunity through induction of DC maturation [2], thus playing important roles in anti-infection, antitumor effect, and autoimmunity.

Autoimmune diseases, including systemic lupus erythematosus (SLE) and rheumatoid arthritis (RA), are characterized by abnormal immune responses to self-antigens. Though the pathogenesis of most autoimmune diseases is not yet fully elucidated, it is generally accepted that they are induced by environmental factors on a genetically susceptible background, leading to abnormality in antigen recognition, antigen presentation, and T/B lymphocyte activation and differentiation, thereby resulting in enhanced production of proinflammatory cytokines and autoantibodies, which eventually cause damage to specific organs and tissues.

Previous studies on *γδ* T cells were mainly concentrated on their anti-infection and antitumor effects, while their roles in the pathogenesis of autoimmune diseases have attracted much attention only in recent years. In this paper, we reviewed the latest knowledge on *γδ* T cells' effects in autoimmune diseases, focusing on SLE and RA, and provide some insight into their possible roles in the pathogenesis of these diseases.

## 2. The Antigen Presenting Function of *γδ* T Cells

Antigen presenting cells (APCs) are necessary for the priming and initiation of antigen-specific T-cell immune responses [3]. Professional APCs mainly refer to dendritic cells (DCs), monocytes/macrophages, and B cells, while nonprofessional APCs include endothelial cells, fibroblasts, and epithelial cells [4]. It has also been shown that *γδ* T cells may function as APCs under certain circumstances.

An *in vitro* study by Brandes et al. showed that when resting blood V*γ*9V*δ*2 T cells were stimulated with IPP, activated V*γ*9V*δ*2 T cells expressed a repertoire of antigen-presentation and costimulation molecules, such as HLA-DR, CD80, CD86, CD40, and CD54. V*γ*9V*δ*2 T cells with such APC-like phenotype could induce strong adaptive responses by primary CD4^+^ and CD8^+^ *αβ* T cells to MHC alloantigens [5]. As V*γ*9V*δ*2 T cells rapidly but transiently upregulate CCR7 upon *γδ*-TCR triggering [6], it may be possible that these V*γ*9V*δ*2 T cells take up and process phosphoantigens in the periphery and then relocate to draining lymph nodes (LNs) where they induce strong adaptive responses in *αβ* T cells. Studies by the same research group revealed that *γδ* T-APCs were more efficient in antigen presentation than monocyte-derived dendritic cells (DCs) [7]. 

As a crucial subset of professional antigen presenting cells, DCs may interact with *γδ* T cells by mutually promoting each other's maturation and function through release of cytokines. A study by Conti et al. showed for the first time that when immature DCs are cocultured with *γδ* T cells activated by phosphoantigens, the expression levels of CD86 and MHC class I molecules on DCs were remarkably upregulated, accompanied by acquisition of functional activities typical of mature DCs [8]. On the other hand, in an *in vitro* culture system, the activation of *γδ* T cells induced by IPP was stronger when DCs were present, indicating a potent costimulating role of DCs on *γδ* T cells [9]. 

Previous studies have confirmed the enhanced capacity of regular APCs, including myeloid DCs (mDCs) and monocytes, on the activation of allogeneic T cells in SLE patients [10, 11]. The abnormal functions of APCs in SLE may be related to downregulation of their cell surface PD-L1 expression, leading to failed antagonization of CD80/CD86-mediated T-cell-signaling transduction and overactivation of effector T cells, thereby contributing to lupus onset [12]. It was also revealed that the number of APCs in the synovial compartment of RA patients is increased, which may activate those effector T cells in the joint and be conducive to the maintenance of synovial inflammation [13]. 

A recent study showed that the peripheral V*γ*9V*δ*2 T cells isolated from RA patients upregulated their expression of APC-specific molecules HLA-DR and CD80/86 when stimulated with IPP *in vitro* and presented soluble antigens and synthetic peptides to CD4^+^ T cells and B cells, thus contributing to sustained activation of CD4^+^ T cells and being associated with RA onset and disease progression [14]. Based on the current data that enhanced APC functions may play a role in the pathogenesis of autoimmune diseases such as SLE and RA by overactivating T and B lymphocytes, it is justified to speculate that the APC-like function of *γδ* T cells may also contribute to the development of these diseases. Affirmative evidence is required to validate the hypothesis. 

## 3. The Proinflammatory Functions of *γδ* T Cells

It has been widely recognized that the abnormal activation of Th1 and Th17 cells and increased production of proinflammatory cytokines such as TNF-*α*, IFN-*γ*, and IL-17 play crucial roles in the pathogenesis of RA. Current studies have shown that in specific microenvironments, *γδ* T cells may display Th1- or Th17-like features, thereby promoting the onset and progression of RA.

*γδ* T cells are able to produce abundant IFN-*γ* and TNF-*α* in the early immune response [15] and are also a critical source of IL-17 in animal models of infectious and autoimmune disorders [16–19]. It was recently reported that the TNF family member CD27 can distinguish between two different subsets of *γδ* T cells: IL-17-producing CD27^−^ *γδ* T cells and IFN-*γ*-producing CD27^+^ *γδ* T cells [20]. Similar to CD4^+^ Th17 cells, the differentiation of which requires several key cytokines including IL-23, IL-21, IL-6, and TGF-*β*, TGF-*β* also plays an essential role in the acquisition of IL-17-producing capacity of *γδ* T cells in the thymus [21]. IL-17-producing *γδ* T cells are CCR6 positive and are maintained by IL-23 [22]. 

Recent studies have validated the role of IL-17 secreted by *γδ* T cells in exacerbation of collagen-induced arthritis (CIA). Roark et al. demonstrated that in both the draining lymph nodes and joints of CIA mice, the vast majority of V*γ*4/V*δ*4^+^ T cells secreted IL-17. Depletion of these cells led to remarkable reduction of clinical disease scores as well as significant decrease in total IgG and IgG2a anticollagen antibodies, suggesting that this subset of V*γ*4/V*δ*4^+^ T cells aggravated CIA by producing IL-17 [23]. The study by Ito et al. also showed that *γδ* T cells were the predominant population in IL-17-producing cells in the inflamed joints of CIA mice, being more abundant than Th17 cells, and the absolute numbers of these cells increased in parallel with disease activity. In contrast, the principal cell type infiltrated in the affected joints of RA patients was Th1 cells while IL-17-producing *γδ* T cells were nearly absent [22]. However, Pöllinger et al. demonstrated that though the number of CD4^+^Th17 cells and IL-17-producing *γδ* T cells in inflicted joints of CIA mice is equal, Th17 cells rather than IL-17^+^ *γδ* T cells play a dominant role in triggering osteoclast-mediated joint destruction [24].

Similar to IL-17, the pathogenic role of TNF-*α* in RA has also been well established. Biologic agents that block TNF-*α* activity are now common in clinical use for RA therapy. Li et al. showed that human peripheral V*γ*9V*δ*2 T cells activated by phosphoantigens could produce TNF-*α* which then cognate with TNF-*α* receptors on these V*γ*9V*δ*2 T cells, thus constituting a positive regulatory mechanism to maintain their responses. These results suggest that TNF-*α* play important roles in the activation and function of human V*δ*2T cells, and TNF-*α* antagonists may affect the function of V*δ*2 T cells and be an important reason for the high risk of tuberculosis in recipients of anti-TNF-*α* therapy [25].

## 4. The Immunoregulatory Effects of *γδ* T Cells

Regulatory T cells (Tregs) play a central role in maintaining the balance between immunity and tolerance, and numerical or functional abnormalities of Tregs are believed to be involved in the pathogenesis of RA, SLE, and other autoimmune diseases. Current data suggests the existence of several Treg populations including CD4^+^CD25^high^Foxp3^+^Treg (sometimes shortly termed as CD4^+^CD25^+^Treg), type 1 T regulatory cells (Tr1), T helper type 3 cells (Th3), and CD8^+^ regulatory T cells (Tcreg) [26, 27]. Based on different sources of generation, Tregs can be divided into thymus-derived natural Tregs (nTreg) and peripheral inducible Tregs (iTreg) by TGF-*β* [26]. Apart from the above-mentioned *αβ* Tregs, recent studies also confirmed the existence of a subset of *γδ* T cells with immunoregulatory functions which may suppress the activity of CD4^+^ T cells and dendritic cells [28]. Earlier studies have indicated that peripheral *γδ* T cells are more capable than CD4^+^Tregs in suppressing the proliferation of CD4^+^ effector T cells; conversely, V*δ*1 T cells displayed stronger inhibitive activity than V*δ*2 T cells in parallel with increased secretion of TGF-*β* [29]. Casetti et al. showed for the first time that a subset of regulatory V*δ*2 T cells expressing Foxp3 could be induced *in vitro* in the presence of specific antigen stimulation and cytokines (TGF-*β*1 plus IL-15) [30].

The role of regular Tregs in the pathogenesis of RA has been extensively investigated. On one hand, it was demonstrated that the number of CD4^+^CD25^high^Tregs was remarkably reduced in peripheral blood of newly onset RA patients [31]. On the other hand, though the frequency of CD4^+^CD25^high^Tregs was significantly higher in the synovial fluid of RA patients than in peripheral blood [32], there existed major functional defects in these Tregs which were unable to suppress the secretion of proinflammatory cytokines such as TNF-*α* by effector T cells [33]. Despite some discrepancy, most studies reported decreased proportions and reduced suppressive capacity of CD4^+^CD25^high^Tregs in active SLE patients as compared with healthy controls [34, 35].

The regulatory functions of *γδ* T cells have been observed in various autoimmune diseases. Consistent with previous observation that *γδ* T cells could express FasL at sites of inflammation and thus induce apoptosis of target cells [36], Ponomarev et al. found that, in the experimental autoimmune encephalomyelitis (EAE) model of the human CNS autoimmune disease multiple sclerosis, *γδ* T cells were able to regulate CNS inflammation and promote disease recovery through Fas/FasL-induced apoptosis of encephalitogenic T cells [19]. More recently, Li et al. found that a subset of CD27^+^CD25^high^ V*δ*1 T cells with immunoregulatory activities expressed Foxp3 and were substantially decreased in the peripheral blood of active SLE patients. Besides, these regulatory *γδ* T cells could be generated *in vitro* under the stimulation with anti-TCR*γδ* in the presence of TGF-*β* and IL-2 [37], suggesting a possible role of regulatory *γδ* T cells in the pathogenesis of SLE. Whether such regulatory *γδ* T cells are present in RA patients and contribute to disease development and progression still needs further research. 

In addition to the regulatory effect exerted by *γδ* T cells *per se*, they can also display immunomodulatory functions through interaction with CD4^+^CD25^+^Tregs. Li et al. first showed that CD4^+^CD25^+^Tregs could significantly inhibit the production of IFN-*γ* by activated *γδ* T cells *in vitro *[38]. *In vivo* studies by Gong et al. using monkeys revealed that activated V*γ*9V*δ*2 T cells by phosphoantigen plus IL-2 could downregulate IL-2-induced expansion of CD4^+^CD25^+^Foxp3^+^Tregs. Consistent with this result, *in vitro* experiments demonstrated that addition of anti-IFN-*γ* antibody led to reduced capacity of activated V*γ*9V*δ*2 T cells to downregulate CD4^+^CD25^+^Foxp3^+^Tregs, suggesting that autocrine IFN-*γ* or its cytokine networks might play a role in the Treg-antagonizing effect of V*γ*9V*δ*2 T cells [39]. Taken together, these results indicate that *γδ* T cells and CD4^+^CD25^+^Tregs have mutual regulatory effect on each other, which may play a part in the pathogenesis of different autoimmune diseases under specific microenvironments.

## 5. The B-Cell Helper Functions of *γδ* T Cells

Studies by Caccamo and colleagues showed that in both the peripheral blood and secondary lymphoid tissues (tonsils) of healthy donors, there exists a subset of CXCR5^+^ V*γ*9V*δ*2 T cells that express the costimulatory markers inducible costimulator (ICOS) and CD40L and produce Th2-type cytokines such as IL-4 and IL-10. Coculture of B-cells with CXCR5^+^ V*γ*9V*δ*2 T cells in the presence of phosphoantigen resulted in an substantial increase in the production of IgG, IgA, and IgM antibodies [40], strongly suggesting that these cells are highly efficient in providing B-cell help for antibody production, an effect similar to that of CD4^+^ follicular B helper T cells (T_FH_) [41]. A recent study by Bansal et al. revealed that human peripheral V*γ*9V*δ*2 T cells activated by phosphoantigen in the presence of IL-21 upregulated their expression of some markers characteristic of T_FH_, including IL-21R, the B-cell attracting chemokine CXCL-13, the CXCL-13 receptor CXCR5, and ICOS, thereby enhancing their potential to promote antibody production by B cells [42]. 

Hyperactivation and dysfunction of B-cells, which ultimately lead to mass production of autoantibodies, play a key role in the pathogenesis of SLE. B-cell depletion for the treatment of SLE has acquired favorable effects [43]. The central role of B cells in the pathogenesis of RA is clear potentially due to biological features including antigen-presenting cells, proinflammatory cytokine, or autoantibody production [44]. Clinical application of B-cell depletion with anti-CD20 monoclonal antibody for the treatment of RA patients results in sustained benefit [45]. Therefore, *γδ* T cells may promote the development of RA, SLE, and other autoimmune diseases by providing help for B cells and enhancing autoantibody production. 

Contrary to this notion, Fujii et al. demonstrated that lupus-prone MRL × C57BL/6 mice lacking *γδ* T cells exhibited more severe disease compared with control mice, suggesting that *γδ* T cells can downregulate disease severity of lupus mice. Further studies showed that *γδ* T cells from one line of MRL/Fas^lpr^ mice GD12 (*γδ*TCR^+^CD4^−^CD8^−^) could kill B cells and inhibit the generation of anti-dsDNA antibodies by *αβ* T-B collaboration *in vitro* in a contact-dependent manner [46]. This discrepant result implied that there might be distinct subsets of *γδ* T cells which act differently on B cells. Direct evidence on the role of *γδ* T cells in regulating autoantibody production in patients or animal models of other autoimmune diseases such as RA is still lacking. 

## 6. Concluding Remarks

**Figure 1:**
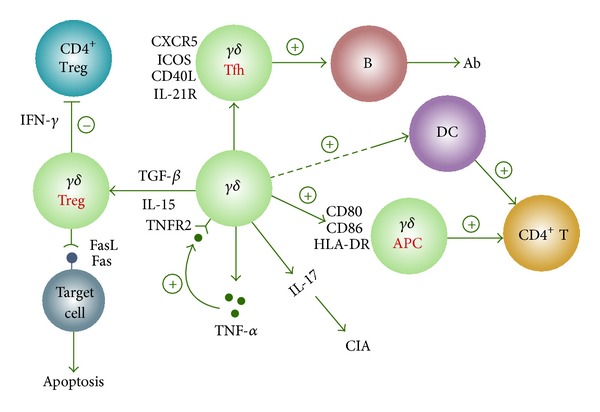
Roles of *γδ* T cells in autoimmune diseases. Ab: antibody, APC: antigen presenting cell, CIA: collagen-induced arthritis, DC: dendritic cell, Tfh: B helper T cell, TNFR2: TNF-*α* receptor 2.

The pathogenesis of most autoimmune diseases, though mainly elusive, is generally ascribed to abnormal immune responses elicited by various environmental factors on a genetically susceptible background, causing production of a large amount of inflammatory cytokines and autoantibodies and eventually leading to disease onset. As a subset of T cells that bridge innate and adaptive immunity, *γδ* T cells may display different functions similar to those of CD4^+^ T-cell subsets such as CTLs, Th1/Th2 cells, Tregs, Th17 cells, and APCs depending on specific microenvironment. They definitely play important roles in the development of autoimmune diseases such as RA and SLE through their antigen-presenting capacity, release of proinflammatory cytokines, immunomodulatory properties, interaction with CD4^+^CD25^+^Tregs, and promotion of antibody production by providing B-cell help (Figure 1). Current data on the role of *γδ* T cells in autoimmune diseases are still scarce; thus in-depth study on their effects in these diseases is of great significance for elucidating the pathogenesis of and developing *γδ* T cell-targeted therapies for these autoimmune diseases.
